# Complete genome of *Pseudomonas putida* strain WBB028 isolated from leaf litter

**DOI:** 10.1128/mra.00234-24

**Published:** 2024-06-11

**Authors:** D. A. Pelletier, W. Alexander, L. H. Burdick, T. A. Rush, J. Tannous, A. B. Webb, J. L. Morrell-Falvey

**Affiliations:** 1 Biosciences Division, Oak Ridge National Laboratory, Oak Ridge, Tennessee, USA; The University of Arizona, Tucson, Arizona, USA

**Keywords:** *Pseudomonas putida*

## Abstract

We report the complete genome of *Pseudomonas putida* strain WBB028, which exhibits broad-spectrum antifungal activity. This strain was isolated from leaf litter collected at Walker Branch Watershed located on the Oak Ridge Reservation in eastern Tennessee (35.9614 N 84.2864 W). The genome is 6.3 Mbp with a 62.5% GC content.

## ANNOUNCEMENT

*Pseudomonas putida* strain WBB028 was isolated from an approximately 2-cm^2^ excised section of leaf litter sample placed on an R2A agar plate incubated at 30°C. Colonies emerged after 2 days and were picked and re-streaked three times on R2A agar until uniform colony morphology was observed. During screening for interactions utilizing a co-culture assay on R2A agar plates with fungal strains isolated from the same sample, WBB028 exhibited antifungal activity against six phylogenetically diverse fungal isolates and was chosen for whole-genome sequencing. *P. putida* strains are metabolically versatile Gram-negative bacteria commonly found in soil (1) or plant associated (2), and more recently, strains have been developed as model systems for metabolic engineering and biotechnology applications (3).

A single colony of strain WBB028 was inoculated in R2A liquid medium and grown overnight at 30°C, with shaking at 200 rpm. DNA extraction was performed on the collected cell pellet using a Qiagen DNAeasy Blood and Tissue Kit (Qiagen, Valencia, CA) per manufacturer’s protocol. DNA was cleaned and concentrated through ethanol precipitation and quantified using an Invitrogen Qubit fluorometer (Thermo Fisher Scientific, Waltham, MA). Approximately 1,000 ng of DNA was sheared using a g-TUBE centrifuged at 500 rpm for a total of 2 min (Covaris, Woburn, MA). Then, size selection was performed to remove fragments under 1,000 bps using 0.4 volumes of HighPrep PCR cleanup beads (MagBio Genomics, Gaithersburg, MD). The recovered DNA was sequenced using a MinION Mk. IIB sequencer and R10.4.1 Flow Cell along with the Rapid Barcoding Kit (SQK-RBK114-24, Oxford Nanopore Technologies, Oxford, UK) following the manufacturer’s instructions. Base calling was performed on an HP Z8 workstation equipped with an NVIDIA RTX A5000 GPU running Guppy v6.2.1+6588110a6 and the High-Accuracy (HAC) base calling model. The sequencing run yielded 17,259 reads with an N50 of 14.3 kbp and a median read quality of 16.9. Prior to assembly, these reads were filtered using Filtlong v0.2.1 to remove reads under 1 kbp and the bottom 5% of reads by quality. Sequence assembly and polishing was performed using Trycycler v0.5.3 (4), along with Flye v2.9 (5), Raven v1.5.3 (6), and Miniasm/Minpolish v0.3-r179 assemblers (7); all software was run using default parameters. The genome was assembled into a single 6,286,687-bp contig with an average read depth of 25x and 62.5% GC.

The genome assembly was annotated using the National Center for Biotechnology Information Prokaryotic Genome Annotation Pipeline (PGAP) (v6.6) (8). The annotation includes 5,478 total CDS, 5,393 protein CDS, 102 RNAs, 22 rRNA genes, (seven complete rRNA operons), four ncRNA, and 76 tRNA genes. Genome completeness and taxonomic assignment were performed using CheckM v1.1.2 and GTDB-Tk v2.3.2, as implemented within KBase (9). Based on the presence of conserved single-copy genes, the genome assembly was estimated to be 100% compete. The average nucleotide identity of its closest relative *P. putida* strain S13.1.2 (10) is 96.0%. The genome will facilitate the investigation of mechanisms of strain WBB028 antifungal activity.
